# The importance of critical incident reporting – and how to do it

**Published:** 2015

**Authors:** Tim Fetherston

**Affiliations:** Consultant ophthalmologist: Sunderland Eye Infirmary, Sunderland, UK. eye999@ymail.com

**Figure:**
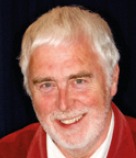
Tim Fetherston

If you asked a group of people whether you were more likely to die from an accident when you were in hospital or when you were travelling, either by air or by car, most people would probably say that it was safer to be in hospital. In fact, this couldn't be further from the truth. If you are a patient, you are a hundred times more likely to die from a critical incident or error in hospital than you are in a transport accident.^1^ Hospitals are dangerous places. Modern treatments are powerful and complex and health care workers face many pressures in terms of workload and funding. In the UK National Health Service (NHS) it is believed that a serious adverse event or critical incident occurs in up to 10% of all hospital admissions. That amounts to about 850,000 adverse events per year^2^ and costs the NHS billions of pounds every year in increased hospital costs, treatments and litigation. The World Health Organization (WHO) estimate that, worldwide, 20–40% of all health care spending is wasted due to poor quality care.

Unfortunately, the health care sector worldwide has been both slow and unimaginative in tackling this huge problem.

Human error, and unsafe procedures and equipment, underlie many of the disasters which occur. Everyone makes mistakes. It is part of being human. Good doctors and good nurses make mistakes, but critical incidents are rarely caused by one person alone.^3^ And yet, traditionally, the response has been to blame those involved and to fail to put systems in place which help to guard against similar problems and errors occurring in the future. All too often, therefore, the same errors have been made repeatedly. This all means that health care staff tend not to report mistakes or ‘near misses’ (errors or disasters that have been narrowly avoided), fearing that if they do so they will be blamed and punished. And this in turn means that senior medical, nursing and management personnel do not get the information they need in order to make the service safer. When the same mistakes occur repeatedly, this is a tragedy, and a gross failure of the care we should deliver for our patients.

**Figure:**
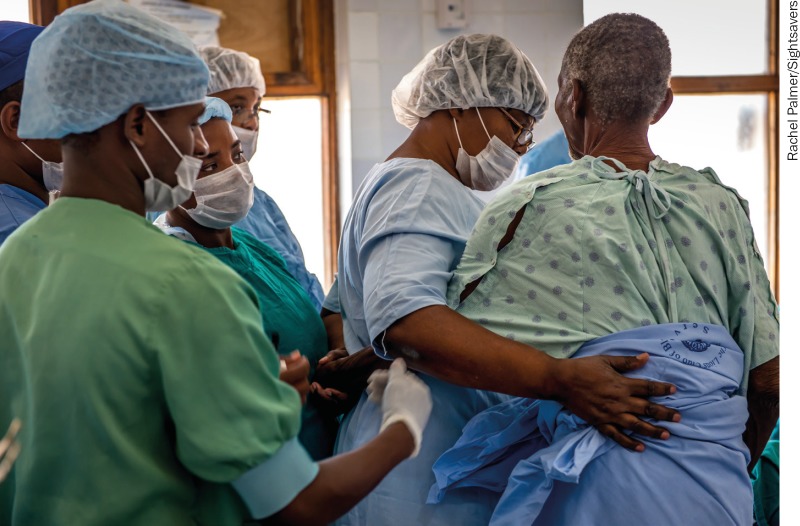
Safety is the responsibility of all staff, no matter how junior or senior they are. MALAWI

The aerospace industry has adopted a fundamentally different approach. For many years, all staff have been encouraged to report problems, failures and mistakes. Safety is the responsibility of all staff, however junior or senior they are, and the culture fosters safety as everyone's first priority. No-one is criticised for reporting a problem – indeed *failure* to report a problem is treated very seriously, and staff have a degree of immunity from any disciplinary action if issues are reported promptly. As a result of this, flying in a commercial airliner is the safest way of travelling, far safer than travelling by car.

**“To err is human, to cover up is unforgivable, and to fail to learn is inexcusable.”**

Prof Liam Donaldson – WHO Envoy for Patient Safety

Although it is impossible to prevent errors, it is possible to put in place procedures which act as barriers to making mistakes. For example, just as airline pilots use a simple checklist when preparing for a flight, an operating theatre checklist can help to ensure that the right patient has the right operation on the right part of the body (page 24). However, if no-one knows what kind of problems are occurring, and how often, it is impossible to design systems which will make health care safer. For example, if there are no reports of drug errors, no-one will know that prescription sheets are confusingly set out. It follows, therefore, that the first, vital, step in improving patient safety is to put in place a completely open system of reporting of all adverse incidents and near misses.

## How to set up an effective reporting system

1. Set up a clinical governance group of senior personnel who are sufficiently experienced to analyse the information and have the authority to make changes in the hospital. The group should have representatives from all relevant departments, and include a senior doctor, a senior nurse, a pharmacist and the hospital manager.
  **Clinical governance group**
  - Senior doctor
  - Senior nurse
  - Hospital manager
  - Pharmacist
  **Information required on an incident reporting form**
  - Patient name and hospital number/date of birth
  - Date and time of incident
  - Location of incident
  - Brief, factual description of incident
  - Name and contact details of any witnesses
  - Harm caused, if any
  - Action taken at the time
  - Name and contact details of the person reporting the incident
2. Design a simple incident reporting form. If the form is long and complicated, people will be reluctant to fill it in.
3. Make sure that the forms are available in each clinical area.
4. Make sure that the completed forms can be sent to the clinical governance group confidentially, so staff members can be confident that the information they provide is kept private.
5. Encourage reporting. This is the difficult part. Because of the culture of blame which has existed for years, staff members may feel they will be victimised if they report incidents. The vital issue is trust. Without trust there is no team, and no teamwork. For the system to work, staff members who report incidents must trust the senior staff and management to treat them justly and not blame them unfairly or make them a scapegoat. Senior staff and management must trust the team to exercise due vigilance, attend training, and to report problems when they occur. Some ways of encouraging reporting are listed in Table 1.

Patient safety is everyone's business. Medical accidents cause suffering to our patients and their relatives, waste huge amounts of money, and are a cause of stress, anxiety and burnout in clinical staff. Improving safety is not a question of ‘trying harder’, but of learning from our mistakes. To do that we need to identify where we go wrong.

**Table 1.** Ways of encouraging reporting of adverse incidents

**Lower the threshold of reporting**	Staff should report even minor incidents and ‘near misses’, which are just as important as major events in identifying and analysing problems with safety.
**Make it clear that the analysis will be looking at all the factors involved, not the actions of one individual**	Training can help to reinforce the concept that incidents rarely have one cause but are almost always multifactorial. All institutional issues should be included in the analysis. It is paramount that everyone understands that patient safety is the business of the whole team.^4^
**Analyse the results logically and formulate an action plan**	Identify the cause of the incident. Focus on the story, and all the contributory issues, not on the individual. Look for all the underlying causes, not just the ‘final error’ which led to the incident. Include the possibility of understaffing, poor design of systems, poor performance, inadequate skill levels, etc. Come up with an action plan which addresses these – perhaps increasing staffing, improving training, improving systems, or using checklists and other protocols to provide barriers to errors. Look for a long-term result, not a short-term fix.
**Feed back the results of the process**	Those who report incidents should be informed of the results of the investigation and the action taken. Key action points should be shared with all clinical staff members. Regular training meetings for all staff members – a team-based approach –should give an outline of some incidents, the problems which lead up to them, and the action taken. Failure to communicate the outcomes to the whole team is cited as a major cause of failure of hospital reporting systems.
**Take action to prevent future incidents**	It will take time for staff members to accept that reporting incidents will not land them in trouble. When they see visible changes, and are made aware that their commitment to safety is valued, most health care workers embrace the reporting system.
**Foster a Team approach**	Make it clear that everyone has a vital role to play. Junior doctors and nurses in particular should be encouraged to contribute, because they may see events and near-misses which more senior staff do not. Senior medical and nursing staff can set an example by completing reports themselves. Ensure that hardworking staff feel valued, and support those who experience stress as a result of being involved in a clinical incident.
